# Babesiosis as a rare cause of fever in the immunocompromised patient: a case report

**DOI:** 10.4076/1757-1626-2-7420

**Published:** 2009-07-10

**Authors:** Daniel A Nelson, Joanna K Bradley, Rajiv Arya, Monica Ianosi-Irimie, Andreia Marques-Baptista, Mark A Merlin

**Affiliations:** Department of Emergency Medicine, University of Medicine and Dentistry of New Jersey, Robert Wood Johnson Medical School1 Robert Wood Johnson Place, MEB Room 104, New Brunswick, NJUSA

## Abstract

**Introduction:**

This is the case of a rare and regional disease not often considered in the immunocompromised patient presenting with a chief complaint of fever.

**Case presentation:**

A 37-year-old immunocompromised Indian woman presented with a chief complaint of fever, in the absence of localizing signs and symptoms, from an area endemic to *Babesia microti*.

**Conclusions:**

Our patient’s case is instructive in that Babesiosis and other arthropod born illnesses should be considered in immunocompromised patients presenting with fever in the absence of localizing signs or symptoms. This is especially true when he or she presents from an area with known endemic disease. While the management of fever in immunocompromised patients is largely standardized, considering Babesiosis from the beginning may prompt early investigation of a blood smear, which has the potential to alert the emergency department physician to Babesiosis. In addition, considering the disease from the outset has the potential to accelerate administration of the appropriate antimicrobial therapy and thus prevent unnecessary morbidity and possible mortality.

## Introduction

The differential diagnosis of fever in the immunocompromised patient is extensive, often including exotic and rare infections. Diagnosis is complicated by the fact that localizing signs and symptoms of infection may be absent in this population. Fever in the immunocompromised patient is most often a result of a community acquired bacterial infection [2]. In addition to infection caused by the more common community acquired bacteria, opportunistic bacterial disease, along with fungal, viral, and arthropod disease, as well as important non-infectious etiologies, need to be considered [12]. In this paper, we present a case of a rare and regional disease not often considered in the immunocompromised patient presenting with a chief complaint of fever.

## Case presentation

A 37-year-old Indian woman presented to our university based, academic emergency department, with a chief complaint of fever of one-week duration. The patient reported the fever was persistent and fluctuated between 102 and 103 degrees Fahrenheit. The elevated temperatures were associated with shaking chills and diffuse myalgias. Review of systems was otherwise unremarkable. She denied any recent change in her appetite or weight.

The patient had a past medical history significant for Systemic Lupus Erythematosis (SLE) that was diagnosed at age 16. She received a cadaveric renal transplant ten years prior to admission secondary to Lupus nephropathy. Over the past year, the patient had suffered from chronic rejection and was on the renal transplant list for a second time. Additional past medical history includes hypertension, chronic *Molluscum contagiosum* on the lower extremities, thyroid cancer status post total thyroidectomy, and renal cell carcinoma status post right native nephrectomy.

Medications included tacrolimus 2 milligrams twice daily, prednisone 5 milligrams daily, darbepoetin alfa 60 milligrams subcutaneously weekly, atenolol 25 milligrams daily, amlodipine 5 milligrams daily, calcitriol 0.25 micrograms daily, levothyroxine 100 micrograms daily, iron supplementation, and a daily multivitamin. Mycophenolate mofetil was recently stopped secondary to epistaxis and gingival bleeding. The patient denied any known drug allergies and was not taking any over the counter medications.

The patient denied any recent travel history but did report spending fourteen days in Nicaragua approximately nine months prior to presentation. While in Nicaragua, the patient recalled a transient diarrheal illness that resolved without incident. The patient denied a history of smoking, alcohol use, illegal drug use, or high-risk sexual behavior.

On physical examination, the patient was awake, alert, and oriented to person, place, and time and in no acute distress. The patient’s vital signs were as follows: blood pressure 112/81, heart rate 115, respiratory rate 16, oral temperature 101.6 degrees Fahrenheit, oxygen saturation on room air 97 percent. The remainder of the patient’s physical examination was unremarkable except for the skin, which was warm, dry, and with numerous 2-5 millimeter pearly, flesh-colored, umbilicated papules on the lower extremities consistent with *Molluscum contagiosum*.

**Figure 1.**
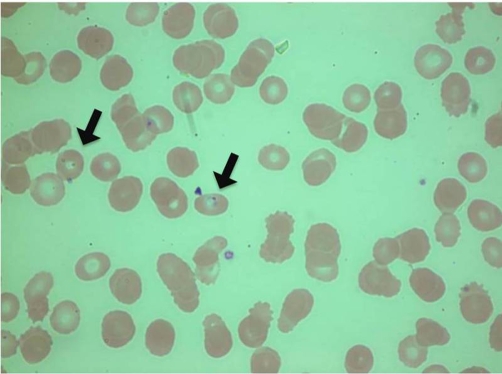
Blood Smear from patient showing intra-erythocyte ring forms.

Initial laboratory studies (Table 1) were significant for a relative monocytosis, normochromic normocytic anemia, increased lactate dehydrogenase with normal Coombs test, thrombocytopenia, high indirect bilirubin, hyponatremia, metabolic acidemia, and renal failure. The patient reported her creatinine was recently measured to be approximately 2.4. It was 3.7 in the emergency department at presentation. A urine analysis was performed and found to contain 1+ blood; however, microscopic analysis failed to reveal any erythrocytes. The patient’s initial chest radiograph was negative for any acute disease process. A blood smear was obtained (Figure 1). Numerous ring trophozites measuring approximately 2.0 microns were seen and estimated to involve 3.8 percent of the erythrocytes. The infected erythrocytes appeared the same size as the surrounding, non-infected cells. No other inclusions, with the exception of the pyroplasms, were seen in the erythrocytes. A rapid malaria antigen test was performed and found to be negative.

**Table 1.** Initial Laboratory Results

Leukocyte Count	4.7 × 10^3^/mm^3^
Hemoglobin	8.1 g/dl
Hematocrit	24 %
Platelet Count	76 × 10^3^/mm^3^
Mean Corpuscular Volume	82.3 μm^3^
Mean Corpuscular Hemoglobin	27.7 pg/cell
Mean Corpuscular Hemoglobin Concentration	33.7 g/dl
Red Cell Distribution Width	14.8 %
Neutrophils	59 %
Band Forms	0 %
Lymphocytes	14 %
Monocytes	26 %
Eosinophils	1 %
Basophils	0 %
	
Sodium	130 mEq/liter
Potassium	4.2 mEq/liter
Chloride	101 mEq/liter
Total Carbon Dioxide	19 mEq/liter
Urea Nitrogen	78 mg/dl
Creatinine	3.7 mg/dl
Glucose	120 mg/dl
Total Protein	6.2 g/dl
Albumin	3.1 g/dl
Total Bilirubin	1.2 mg/dl
Direct Bilirubin	0.3 mg/dl
Alkaline Phosphatase	53 U/liter
Aspartate Aminotransferase	38 U/liter
Alanine Aminotransferase	32 U/liter
	
Blood Culture 1	neg
Blood Culture 2	neg
Urine Culture	neg

At this point, the patient was admitted with a presumptive diagnosis of Babesiosis. The patient was started on azithromycin 250 milligrams daily, doxycycline 100 milligrams twice daily, and atovaquone 750 milligrams twice daily. On hospital day two, additional data was obtained about the patient’s hematologic status. The patient was found to have a reticulocyte count of 1.32 (an inadequate bone marrow response most likely secondary to chronic renal failure), a haptoglobin level less than 7, and a falling hemoglobin level. In the setting of increased red cell destruction and decreased red cell production, the patient was transfused two units of packed red blood cells. The patient responded well to the transfusions and her hemoglobin subsequently stabilized while her lactate dehydrogenase and haptoglobin improved only marginally.

While the patient’s hemoglobin initially dropped, the patient’s renal function steadily improved with treatment of her underlying infection. By the end of the patient’s one-week hospital stay, her creatinine had returned to baseline. In addition, her bilirubin, electrolytes, white blood cell count, and platelet count improved over the week.

The diagnosis of Babesiosis was eventually confirmed by polymerase chain reaction (PCR) performed by Focus Diagnostics Incorporated in Cypress, California. In addition, a diagnosis of Lyme disease was presumed secondary to elevated Lyme titers and high rate of concurrent infection of Babesiosis with Lyme disease. Our patient did extremely well and was discharged home with no residual symptoms.

## Discussion

Babesiosis was first discovered at the end of the 19th century as a cause of “red water fever” in cattle [1]. Later, *Babesia* became the first recognized arthropod-borne pathogen of vertebrates [5]. Today, it is a known zoonotic cause of human febrile disease. The etiologic agent of Babesiosis in North America is most often *Babesia microti*, a protozoan parasite of small mammals. *Babesia divergens* is found as the etiologic agent of Babesiosis in Europe. *Babesia* is transmitted by the *Ixodes scapularis*, more commonly known as the deer tick. This same tick is also responsible for the transmission of *Borrelia burgdorferi*, the etiologic agent of Lyme disease, and *Anaplasma phagocytophila*, the etiologic agent of human granulocytic anaplasmosis. Humans most often acquire the disease through the bite of the deer tick and infrequently through contaminated blood transfusions [3].

According to the Centers for Disease Control, *Babesia* is endemic to the Northeastern United States, more specifically New York, Massachusetts, Connecticut, and Rhode Island. There is also a considerable focus of disease in Wisconsin and Minnesota. In 2003, New Jersey was added to the list of states *Babesia* calls home. All reported and confirmed cases of Babesiosis in New Jersey have occurred in the central portion of the state with most being reported in Burlington and Ocean Counties [4]. Peak transmission occurs from May to September, with July being the most common month of infection. Children and adults are affected equally; however, adults tend to have a greater proportion of symptomatic infections [5].

Clinically, Babesiosis most often presents in a flu-like manner with fever, chills, diaphoresis, malaise, myalgias, and arthralgias. Patients have also reported headache, neck stiffness, sore throat, cough, shortness of breath, anorexia, nausea, vomiting, and hepatosplenomegaly. Patients can also be completely asymptomatic. In a study by Hatcher et al of thirty-four cases of Babesiosis in an endemic area of New York, it was found that patients presented on average 15.4 days after the onset of symptoms. In addition, only thirty-two percent of patients were able to recount a tick bite [3].

Objectively, patients often present with hemolytic anemia as evidenced by an elevated indirect bilirubin, elevated lactate dehydrogenase, and depleted haptoglobin levels. If hemolysis is present, urine analysis often reveals hemoglobinuria and proteinuria without the concomitant presence of microscopic red cells. Leucopenia and thrombocytopenia may also be present secondary to a Tumor necrosis factor (TNF)-mediated immune response. Patients may also have elevated liver enzymes including aspartate aminotransferase, alanine aminotransferase, and alkaline phosphatase [5].

Babesiosis can often be clinically confused with Ehrlichiosis, Lyme disease, Malaria, Rocky Mountain Spotted Fever (RMSF), and Typhoid. When the history and physical are inadequate to make a diagnosis, as in the absence of a telltale “bulls eye” or centripetal rash, distinguishing the aforementioned diseases can start with an investigation for the presence or absence of hemolytic anemia as outlined above. Doing so would reliably rule out Ehrlichiosis, Lyme disease, RMSF, and Typhoid, as hemolytic anemia is uncharacteristic of these diseases. However, it is important to note that concurrent infection of Babesiosis with Lyme disease and Ehrlichiosis has been reported [8].

Differentiating *Plasmodium*, the etiologic agent of Malaria, from *Babesia*, the etiologic agent of Babesiosis, can be achieved in a number of ways. The simplest is through giemsa-stained thin blood smears revealing intra-erythrocyte ring forms, also known as pyriform inclusions, with light blue cytoplasm. A smear of *Babesia* infected erythrocytes is characteristically very similar to that of erythrocytes infected with *Plasmodium*; however, they can be differentiated by the greater degree of pleomorphisms as well as the presence of the pathognomonic finding of trophozites in the form of Maltese crosses in *Babesia* smears. Diagnosis is confirmed through serologic testing or PCR. PCR targeting of the 18S rDNA portion of the genome is more sensitive than, and equally specific as, serologic testing in the detection of acute *Babesia spp*. Infections with as few as three parasites in 100 microliters of blood can yield a positive result with PCR [5,6].

Microscopically, *Babesia* invades erythrocytes and causes damage through parasite directed alterations of the erythrocyte membrane [9]. These changes cause erythrocytes to adhere to the endothelium of the microvasculature resulting in excessive pro-inflammatory cytokine release and tissue hypoxia [5]. In addition, the finding of anemia is a result of the lysis of erythrocytes [10]. The severity of disease is proportional to the parasite load with complications most commonly occurring in those individuals with a parasitemia level greater than 10.0 percent or hemoglobin less than 10.0 grams/deciliter on admission [3]. Complications of Babesiosis include acute respiratory failure (20.6%), disseminated intravascular coagulation (17.6%), non-cardiogenic pulmonary edema (11.8%), and renal failure (5.9%) [3].

Immunocompromised hosts are more susceptible to Babesiosis than the immunocompetent population. More specifically, those patients treated with prednisone and tacrolimus are at risk of symptomatic infection because these drugs suppress T-cell activation and calcineuron/interleukin-2 production needed to fight an intracellular pathogen. Because of this decreased immune response, an immunocompromised patient is more likely to have a high peak parasitemia level and thus more likely to suffer from severe disease and complications [3,7,9].

The rate of mortality has been estimated to be approximately five percent in patients with clinically evident disease and thus treatment is indicated for all patients presenting for medical care. Prior to 2000, the treatment of Babesiosis had classically entailed a combination of quinine and clindamycin. In 2000, Krause et al showed that the combination of atovaquone and azithromycin was as efficacious as the standard treatment and considerably more tolerable. Patients with severe disease and those at risk for complications may also require exchange transfusion which may be curative in those situations. Interestingly, *Babesia* infections in immunocompromised individuals can persist despite treatment. These patients are more likely to relapse or continue to remain subclinical hosts for the parasite. In 2008, Krause et al found that immunocompromised patients required treatment for at least two weeks after the blood smear no longer shows trophozites in erythrocytes in order for a cure to have been achieved [9-11].

## Conclusions

Our patient’s case is instructive in that Babesiosis and other arthropod born illnesses should be considered in immunocompromised patients presenting with fever in the absence of localizing signs or symptoms. This is especially true when he or she presents from an area with endemic disease. While the management of fever in immunocompromised patients is largely standardized from an emergency department perspective, considering Babesiosis from the beginning may prompt early investigation of a blood smear, which has the potential to alert the emergency department physician to Babesiosis. In addition, considering the disease from the outset has the potential to accelerate administration of the appropriate antimicrobial therapy and thus prevent unnecessary morbidity and possible mortality.

## List of abbreviations

PCR: Polymerase Chain Reaction
pg/cell: Picograms/cell
RMSF: Rocky Mountain Spotted Fever
SLE: Systemic Lupus Erythematosis
TNF: Tumor Necrosis Factor
